# The influence of menstrual cycle and endometriosis on endometrial methylome

**DOI:** 10.1186/s13148-015-0168-z

**Published:** 2016-01-12

**Authors:** Merli Saare, Vijayachitra Modhukur, Marina Suhorutshenko, Balaji Rajashekar, Kadri Rekker, Deniss Sõritsa, Helle Karro, Pille Soplepmann, Andrei Sõritsa, Cecilia M. Lindgren, Nilufer Rahmioglu, Alexander Drong, Christian M. Becker, Krina T. Zondervan, Andres Salumets, Maire Peters

**Affiliations:** Competence Centre on Health Technologies Tartu, Tartu, Estonia; Tartu University Women’s Clinic, Tartu, Estonia; Institute of Bio- and Translational Medicine, University of Tartu, Tartu, Estonia; Institute of Computer Science, University of Tartu, Tartu, Estonia; Elite Clinic, Tartu, Estonia; Women’s Clinic, Tartu University Hospital, Tartu, Estonia; Wellcome Trust Centre for Human Genetics, University of Oxford, Oxford, UK; Endometriosis CaRe Centre, Nuffield Department of Obstetrics & Gynaecology, University of Oxford, Oxford, UK

**Keywords:** DNA methylation, Endometriosis, Endometrium, Epigenetics, Illumina 450K, Menstrual cycle, Microarray

## Abstract

**Background:**

Alterations in endometrial DNA methylation profile have been proposed as one potential mechanism initiating the development of endometriosis. However, the normal endometrial methylome is influenced by the cyclic hormonal changes, and the menstrual cycle phase-dependent epigenetic signature should be considered when studying endometrial disorders. So far, no studies have been performed to evaluate the menstrual cycle influences and endometriosis-specific endometrial methylation pattern at the same time.

**Results:**

Infinium HumanMethylation 450K BeadChip arrays were used to explore DNA methylation profiles of endometrial tissues from various menstrual cycle phases from 31 patients with endometriosis and 24 healthy women. The DNA methylation profile of patients and controls was highly similar and only 28 differentially methylated regions (DMRs) between patients and controls were found. However, the overall magnitude of the methylation differences between patients and controls was rather small (Δβ ranging from –0.01 to –0.16 and from 0.01 to 0.08, respectively, for hypo- and hypermethylated CpGs). Unsupervised hierarchical clustering of the methylation data divided endometrial samples based on the menstrual cycle phase rather than diseased/non-diseased status. Further analysis revealed a number of menstrual cycle phase-specific epigenetic changes with largest changes occurring during the late-secretory and menstrual phases when substantial rearrangements of endometrial tissue take place. Comparison of cycle phase- and endometriosis-specific methylation profile changes revealed that 13 out of 28 endometriosis-specific DMRs were present in both datasets.

**Conclusions:**

The results of our study accentuate the importance of considering normal cyclic epigenetic changes in studies investigating endometrium-related disease-specific methylation patterns.

**Electronic supplementary material:**

The online version of this article (doi:10.1186/s13148-015-0168-z) contains supplementary material, which is available to authorized users.

## Background

DNA methylation, an important epigenetic mechanism crucial for maintaining tissue-specific gene expression pattern [1, 2], is suggested to be one possible molecular feature that contributes to the development of many human diseases, including endometriosis. Deviation from normal DNA methylation level may lead to alterations in the cellular microenvironment, affect gene expression and initiate pathologic processes. During the last decade, several studies have reported abnormal methylation patterns of selected genes, e.g. steroidogenic factor 1 [3], progesterone receptor B [4], oestrogen receptor-β [5], HOXA10 [6–8], HOXA11 [9], COX-2 [10] and aromatase [11], in endometriotic lesions and endometria of endometriosis patients. Advancements in microarray technology have now allowed to assess DNA methylation on a global scale; and to date, already four studies, though relatively small and using different array platforms, have suggested genome-wide differences between endometriosis patients’ endometria and lesions [12–14] or between endometrial tissues of patients and controls [15]. Studies on endometriotic lesions or stromal cells originating from lesions revealed clear evidence of epigenetic alterations that could be associated with the disease [12–14]. The issue whether the primary source of these alterations is endometrial tissue or epigenetic alterations occur during the formation of lesions in abdominal cavity in response to changed abdominal environment has been addressed in the study by Naqvi et al. [15], who evaluated endometrial DNA methylation profile of patients and controls and suggested that some epigenetic alterations occur already in the endometria of endometriosis patients. Furthermore, it has been suggested that alterations on genetic and epigenetic levels during early embryogenesis may lead to endometriosis development because the fine-tuning mechanisms responsible for the correct development of the female genital system are disrupted [16, 17].

Endometrium is a unique tissue undergoing cyclic breakdown and regeneration, and similarly to other tissues and cell types [18, 19], has its own distinct DNA methylation pattern that is influenced by cyclic hormonal changes [20]. The menstrual cycle phases of the studied women were not shown in a previous study examining endometrial methylome of endometriosis patients [15]; however, in the light of the recent knowledge about the significant impact of menstrual cycle phases on the endometrial methylome of healthy women [20], it is apparent that normal cyclic epigenetic signature of endometrial tissue should be considered when studying endometrial tissue-related disorders, like endometriosis.

During the past 10 years, a number of studies have been conducted to find reliable diagnostic biomarkers for endometriosis, unfortunately with little success. The need for non-invasive or minimally invasive biomarkers is difficult to underestimate as the average delay between the onset of symptoms and the surgical diagnosis is almost 7 years [21]. Such biomarkers would enable to avoid the unnecessary laparoscopy while endometriosis is suspected but not present and make possible to get the right diagnosis of endometriosis much earlier. Therefore, the aim of the current study was to reveal potential epigenetic biomarkers from endometrial DNA of endometriosis patients’ endometria, from endometriosis centres in Tartu (Estonia) and Oxford (UK), by considering the menstrual cycle dependent changes. Furthermore, we aimed to investigate the menstrual cycle-specific methylation signature to widen the knowledge about the epigenetic changes occurring during endometrial growth across the entire menstrual cycle.

## Results

### Genome-wide DNA methylation analysis of endometrial tissues from patients with endometriosis and healthy women

Endometrial samples from 31 endometriosis patients and 24 disease-free women, from menstrual (M, *n* = 5), proliferative (P, *n* = 5), early-secretory (ES, *n* = 8), mid-secretory (MS, *n* = 26) and late-secretory (LS, *n* = 11) menstrual cycle phases (Table 1), were used for genome-wide DNA methylation analysis.

**Table 1:** General characteristics of the study participants

Microarray study	Patients with endometriosis (*n* = 31)		Disease-free women (*n* = 24)	
	Estonian patients	Oxford patients	Estonian controls	Oxford controls
	(*n* = 24)	(*n* = 7)	(*n* = 17)	(*n* = 7)
Age (years ± SD)	31.0 ± 4.0	36.0 ± 5.0	30.1 ± 3.2	34.2 ± 6.2
BMI (mean, kg/m^2^ ± SD)	21.8 ± 3.1	23.6 ± 2.0	23.6 ± 4.2	26.0 ± 4.3
Smoking (*n*)	0	2	0	0
Stage I–II (*n*)	16	3	NA	NA
Stage III–IV (*n*)	8	4	NA	NA
Only endometrioma (*n*)	0	4	NA	NA
Only peritoneal lesions (*n*)	14	3	NA	NA
Peritoneal lesions together with endometrioma (*n*)	10	0	NA	NA
Menstrual cycle characteristics				
Menstrual phase (days 1–5), (*n*)	0	4	0	1
Proliferative phase (days 6–14), (*n*)	0	2	0	3
Early-secretory phase (days 15–20),(*n*)	7	0	0	1
Mid-secretory phase (days 21–23), (*n*)	8	1	17	0
Late-secretory phase (days 24–28), (*n*)	9	0	0	2
Validation study	Patients with endometriosis (*n* = 15)		Disease-free women (*n* = 14)	
Age (years ± SD)	31.0 ± 3.39		32.0 ± 2.7	
BMI (mean, kg/m^2^ ± SD)	20.0 ± 3.92		23.1 ± 5.73	
Smoking (*n*)	1		2	
Mid-secretory phase (days 21–23), (*n*)	7		7	
Late-secretory phase (days 24–28), (*n*)	8		7	
Stage I–II (*n*)	13		NA	
Stage III–IV (*n*)	2		NA	

*NA* not applicable

**Fig. 1:**
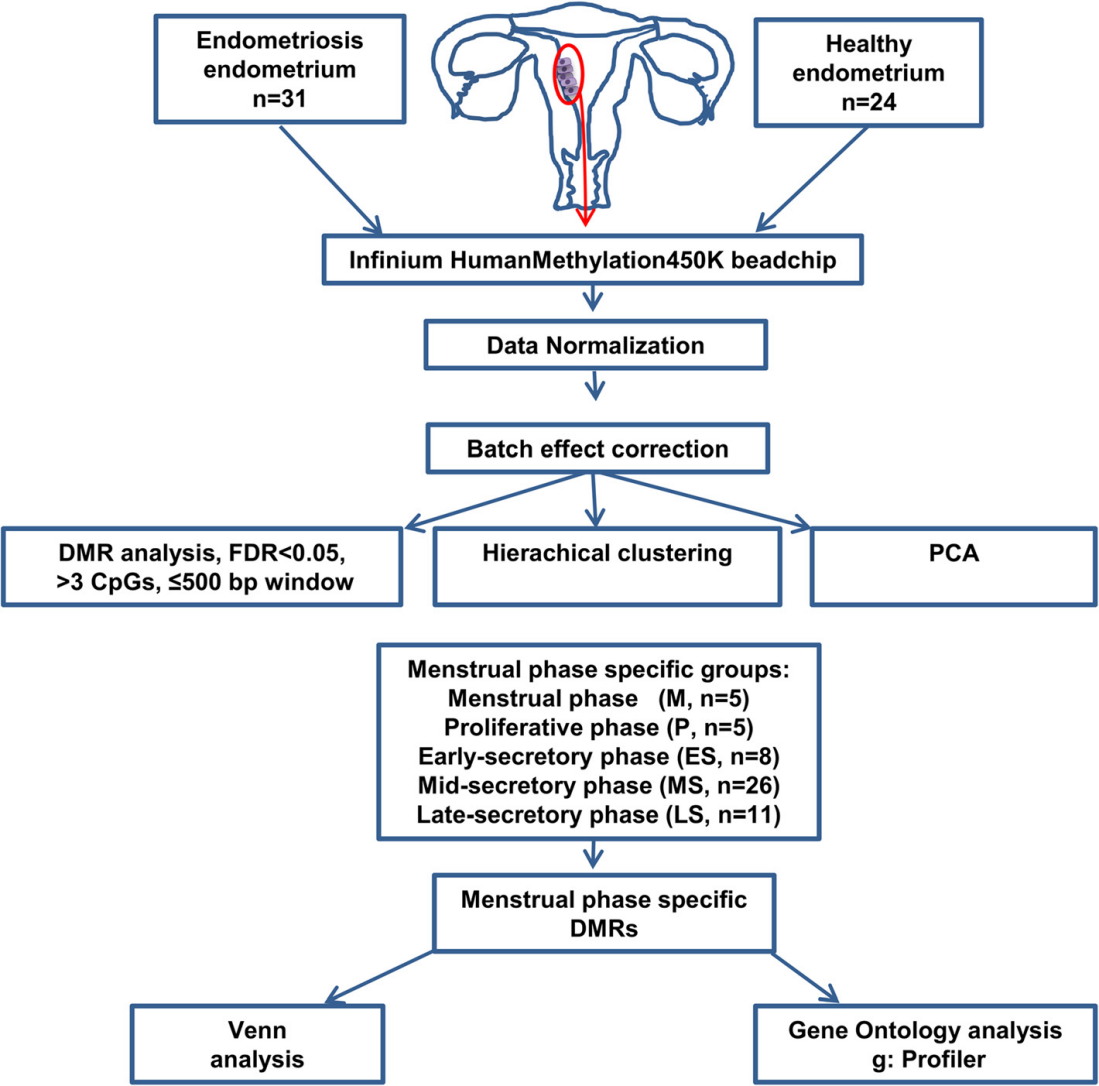
Schematic representation of the study design and main steps of the data analysis

The pipeline of the study is given in Fig. 1. Principal component analysis (PCA) clustering of the normalised data was used to describe the endometrial DNA methylation profiles of patients with endometriosis and healthy women (Additional file 1). Approximately 19.6 % of variation across all studied probes was accounted for in the first two principal components (12.4 % for PC1 and 7.2 % for PC2), and no significant segregation between patients and controls was noticed, indicating that the overall DNA methylation profile between patients and controls was very similar. Still, if we compared the methylation profiles of all patients with endometriosis to healthy women, we found 28 differentially methylated regions (DMRs) (false discovery rate, FDR <0.05, Δβ ranging from –0.01 to –0.16 and from 0.01 to 0.08) from which 16 were associated to known genes (*PI3*, *SLC43A3*, *MGAT5B*, *MUC4*, *HIVEP3*, *FGG*, *CLCF1*, *CANT1*, *LTK*, *AHRR*, *AKR1B1*, *APEH*, *CST11*, *ELOVL4*, *HBE1* and *NEGR*1) (Additional file 2). One of the top-ranking intergenic DMRs was located on chromosome locus 7p15.2, about 13 kb upstream from *HOXA* gene cluster.

**Fig. 2:**
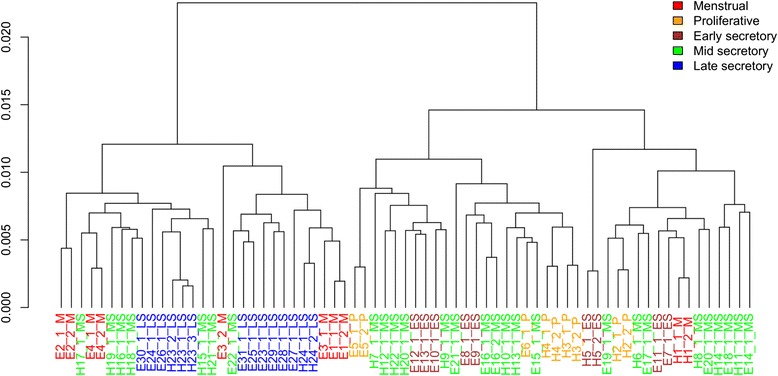
Hierarchical clustering analysis of all endometrial samples included into the study. Sample codes starting with *E* indicate patients with endometriosis and *H* indicates healthy individuals. Samples with the same index number are duplicates

Unsupervised hierarchical clustering of the same data (Fig. 2) revealed two main branches that divided endometrial samples based on the menstrual cycle phase rather than diseased/non-diseased status. The first branch included all LS phase samples (*n* = 11), four out of five M phase (*n* = 4) and some MS phase (*n* = 7) samples, while the other branch included the majority of samples from MS (*n* = 19) phase, ES phase (*n* = 8), P phase (*n* = 5) and one remaining sample from M phase. Therefore, to consider the impact of menstrual cycle on endometriosis-specific methylation signature, we determined the differences associated with menstrual cycle phases.

### Menstrual cycle-specific DNA methylation signature and gene ontology (GO) analysis of differentially methylated regions

As unsupervised hierarchical clustering analysis revealed no segregation between patients and controls, both groups were combined (altogether 55 individuals) to find menstrual cycle phase-specific methylation changes. The studied individuals were divided into five groups according to the menstrual cycle day at the time of biopsy collection: (1) M (*n* = 5), (2) P (*n* = 5), (3) ES (*n* = 8), (4) MS (*n* = 26), and (5) LS phase (*n* = 11) groups. To assess the overall methylation pattern characteristic to each cycle phase, the methylation data of each phase was compared to other phases. A large number of differentially hypo- and hypermethylated regions (FDR < 0.05) were noticed when either or both M and LS phases were involved in comparisons, while only some DMRs were found in comparisons between P, ES and MS phases (Table 2, Additional file 3).

**Table 2:** The number of differentially hyper- and hypomethylated DMRs and genes between menstrual cycle phases

	DMR and genes (hyper-/hypomethylated)	M (*n* = 5)	P (*n* = 5)	ES (*n* = 8)	MS (*n* = 26)	LS (*n* = 11)
M (*n* = 5)	DMR		1009/1775	130/189	363/855	288/368
	Genes		632/1066	92/116	254/512	208/222
P (*n* = 5)	DMR	1009/1775		0/0	1/2	3045/1650
	Genes	632/1066		0/0	1/0	1768/936
ES (*n* = 8)	DMR	130/189	0/0		0/5	2806/1208
	Genes	92/116	0/0		0/3	1015/635
MS (*n* = 26)	DMR	363/855	1/2	0/5		2806/1208
	Genes	254/512	1/0	0/3		1616/704
LS (*n* = 11)	DMR	288/368	3045/1650	1727/1050	2806/1208	
	Genes	208/222	1768/936	1015/635	1616/704	

*DMR* differentially methylated regions, *M* menstrual phase, *P* proliferative phase, *ES* early-secretory phase, *MS* mid-secretory phase, *LS* late-secretory phase

As the endometrial tissue growth and degradation during the menstrual cycle is a continuum where 1-cycle phase progresses to another, only genes that were differentially methylated in adjacent phases (M vs. P, MS vs. LS and LS vs. M) were included in the downstream analysis. The complete lists of DMRs were subjected to enrichment analysis that revealed significant enrichment for multiple ontology terms (the lists of Gene Ontology—GO terms and Kyoto Encyclopaedia of Genes and Genomes—KEGG pathway analysis are outlined in Additional file 4 and 5).

**Fig. 3:**
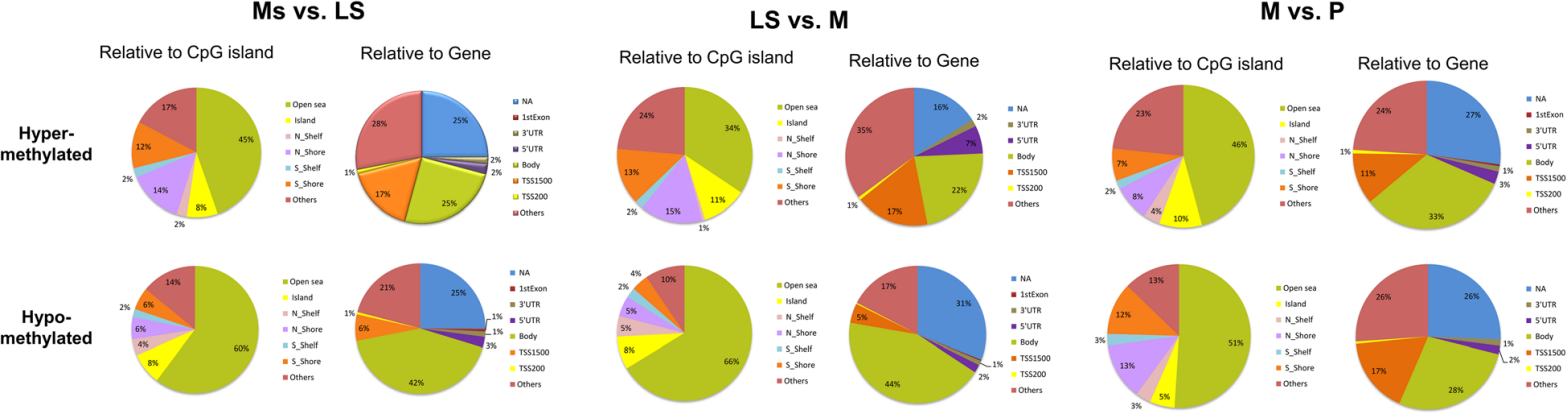
Pie charts of DMRs between different menstrual cycle phases in relation to CpG island and relative to gene. CpG content together with neighbourhood context was defined as (i) open sea; (ii) island—DNA sequence at least 200 bp and GC content greater than 50 %, island shores—2 kb regions upstream and downstream of the CpG islands and shelves—2 kb regions upstream and downstream of the CpG island shores and (iii) others (DMRs with several annotations). Gene context was defined as promoter region (TSS1500—201 to 1500 bp upstream of transcription start site, TSS200—200 bp to transcription start site and 5′ UTR), the 1st exon of transcript; the gene body; 3′ UTR and NA—non-island and others (DMRs with several annotations)

The CpG island (DNA sequence at least 200 bp and GC content greater than 50 %) hypermethylation in gene promoter regions has been associated with repression of gene transcription and hypermethylation of regions next to CpG islands, island shores (2 kb regions upstream and downstream of the CpG islands) and shelves (4 kb regions upstream and downstream of the CpG islands) with higher gene expression [22]. Therefore, the location of the differentially methylated CpG sites in relation to genomic elements such as CpG islands, island shores and shelves, open sea (all remaining sequence) and gene structure (promoter region, 5′ UTR, first exon, gene body, 3′ UTR and intergenic) was analysed to investigate differential representation of functional categories between different menstrual cycle phases (Fig. 3). The assessment of distribution of hypo- and hypermethylated DMRs showed slight overrepresentation of CpGs located in the open sea (ranging from 34–66 %) compared to CpGs located within and next to islands (island, shores and shelves, ranging from 24–42 %), when the CpG distribution relative to CpG islands was analysed. The lowest number of CpGs was seen in shelves (ranging from 4–7 %), whereas higher number of CpG sites was located within CpG islands (ranging from 5–11 %) and the highest number of CpG sites was located in shores (ranging from 9–28 %). When the distribution of CpGs in relation to genes was examined, it was evident that large proportions of CpGs were located in intergenic regions and gene bodies (ranging from 38–75 %) and only a minority of CpGs (ranging from 8–25 %) were in gene promoter areas. However, when enrichment analysis of DMRs based on their location (promoter/gene body) was carried out, no GO terms or KEGG pathways characteristic to specific menstrual cycle phase were found.

Next, the complete lists of differentially methylated genes from each cycle phase comparisons were subjected to Venn analysis to reveal genes characteristic to specific menstrual cycle phases (Additional file 6). The results showed 5 hypo- and 5 hypermethylated genes for M phase and 127 hypo- and 113 hypermethylated genes for LS phase (central intersection in the Venn diagram, gene lists are given in Additional file 7) but no enrichment of specific GO terms or KEGG pathways was found.

### Differentially methylated genes between patients and controls—effect of menstrual cycle phases

As menstrual cycle phase comparisons revealed several differences in the methylation pattern, we compared the lists of endometriosis-specific differentially methylated genes and regions to the menstrual cycle-specific alterations. Results showed that eight out of 16 differentially methylated genes found in patients with endometriosis overlapped with the menstrual cycle-related genes: seven genes (*PI3*, *SLC43A3*, *MGAT5B*, *MUC4*, *HIVEP3*, *FGG* and *CANT1*) from comparison between MS and LS phases and one gene (*LTK*) from M to P comparison. The remaining eight differentially methylated genes—*AHRR*, *AKR1B1*, *APEH*, *CST11*, *ELOVL4*, *CLCF1*, *HBE1* and *NEGR*1 were not related to the menstrual cycle changes. From DMRs that were not related to any genes, five were also found in the lists of menstrual cycle-specific genes. However, the top-ranking DMR near the *HOXA* gene cluster was not found to be associated with any specific menstrual cycle phase. To eliminate all potential confounders that may come from menstrual cycle phase differences, we also compared patients and controls only from MS phase group because this was the group with the largest number of individuals (8 patients vs. 17 controls) in our dataset. Interestingly, the MS phase group analysis revealed no DMRs.

### Validation of methylation data by direct bisulfite sequencing

To confirm the results of microarray analysis, four CpG sites located in the promoter regions (two CpGs from *CST11* gene, one from *PI3* gene and one from *SLC43A3* gene) with differential methylation between patients with endometriosis and healthy women were selected for validation analysis by conventional bisulphite Sanger sequencing in an extended group of patients and controls from LS (*n* = 15) and MS phase (*n* = 14). The correlation analysis between microarray and bisulphite sequencing data showed strong correlation (Pearson’s correlation coefficient, PCC > 0.85, *P* < 0.001) for *SLC43A3* and *CST11* and moderate correlation (PCC = 0.58, *P* = 0.07) for *PI3*. From four analysed CpG sites, only the CpG from *SLC43A3* gene showed statistically significant differential methylation between MS patients and controls (*P* = 0.03).

## Discussion

To the best of our knowledge, this is the first study assessing the methylome of endometria of endometriosis patients and controls using Infinium HumanMethylation 450K BeadChip array and taking into account DNA methylation changes during the menstrual cycle. The results of this study suggest that overall endometrial DNA methylation signature is highly similar between patients with endometriosis and healthy women but largely influenced by the menstrual cycle phases. Additionally, our study describes normal endometrial methylome throughout the menstrual cycle and shows that the largest changes in epigenetic signature occur in late-secretory and menstrual phases.

The usability of epigenetic biomarkers in clinical setting has been accepted and new and simple methodologies allowing straightforward DNA methylation biomarker detection in routine diagnostics have already been developed [23]. Previous endometriosis studies have provided evidence that the epigenetic changes not only occur in ectopic endometriotic lesions but are already present in the eutopic endometrium of endometriosis patients [15]. Therefore, the combination of eutopic endometrium that is easily obtainable by the semi-invasive sampling procedure and assessment of DNA methylation markers could offer an excellent source for epigenetic biomarker discovery. So far, four microarray-based studies in eutopic endometria [15], eutopic/ectopic endometria [12] and primary stromal cell cultures of eutopic and/or ectopic endometria [13, 14] have been performed focusing rather on disease pathogenesis than on clinical usability. Only one study concentrated on eutopic endometria [15] in the perspective of using epigenetic markers as potential targets for therapeutic agents. Despite finding a large number of differentially methylated genes, the authors concluded that methylation and demethylation are both common events in endometrium, making the broad use of therapeutics affecting the methylation level impractical [15]. In our study, we tried to find endometrial epigenetic markers useful for diagnostic purposes. However, the results of our study indicated that endometrial tissue epigenetic signature in patients and controls is highly similar and only a few DMRs were found, indicating that alterations in endometrial methylation pattern are not common in endometriosis. The lack of substantial differences in endometrial epigenetic signature in endometriosis was proposed also by another study [13], where cultured primary stromal cells from eutopic and ectopic endometria of endometriosis patients and healthy women were used. Therefore, we suggest that endometrial DNA methylation differences do not provide good biomarkers with acceptable sensitivity and specificity for discrimination of patients with endometriosis and healthy women.

The further validation of selected CpGs in extended subsets of patients and controls from MS and LS phase confirmed differential methylation of CpG in *SLC43A3* promoter, however, only between the MS patients and controls. The *SLC43A3* hypermethylation was noticed in MS phase in our menstrual phase study and also while all patients were compared to controls, indicating influence both from disease and menstrual cycle phase. Our results are supported by the study conducted by Tamaresis et al. [24] who found that several genes showing differential methylation in our study (such as *AHRR*, *APEH*, *ELOV4*, *PI3*, S*LC43A3*, *MUC4*, *CANT* and *CLCF1*) revealed also differential expression between patients and controls from certain menstrual cycle phases. Interestingly, *SLC43A3* was found to be differentially expressed only between MS phase patients and controls suggesting that small-scale methylation alterations can probably affect the expression of this gene. There is only some data about the function of *SLC43A3*, but very recently, it was proposed that *SLC43A3* is a purine-selective nucleobase transporter [25]. *SLC43A3* is expressed during embryogenesis [26] but the possible role in endometrium or endometriosis development remains to be elucidated.

There is an evidence both on transcriptome [27, 28] and epigenome levels [20] that endometrial molecular signature is largely influenced by the menstrual cycle. The significant impact of menstrual cycle phases to overall endometrial methylome was confirmed also in our menstrual cycle phase-specific analysis where we saw that major epigenetic changes occurred while MS phase turned to LS phase, by which point, the endometrial tissue has reached its maximal thickness and secretory capacity, predecidual changes begin in the stroma and the endometrium is ready for embryo implantation. However, if no implantation takes place, the degradation processes are initiated. Also, significant changes were found between LS and M phases, when the desquamation of the tissue is followed by endometrial shedding and menstruation, and between M and P phases, when active repair and regeneration processes in endometrial tissue are taking place. In light of these results, it is evident that normal endometrial methylation level fluctuations during the menstrual cycle should be taken into account while searching endometrial biomarkers.

However, we believe that the relevance of epigenetic markers in the context of disease pathogenesis or menstrual cycle biology cannot be underestimated. For instance, one of the most statistically significantly hypomethylated DMRs in patients was an intergenic CpG island about 13 kb upstream from the *HOXA* gene cluster. Whether small but statistically significant differences in methylation levels could affect gene expression levels is currently unknown, but previous studies have shown that the members of *HOXA* cluster, *HOXA10* and *HOXA11* were differentially methylated in stromal cells obtained from endometriomas [14] and in eutopic endometria from patients [6, 7, 9, 29, 30] compared to healthy controls, and hypermethylation of the HOXA genes was accompanied by lower transcript and protein levels in endometrium of endometriosis patients [6, 9]. In a recent review, Kobayashi et al. [31] assessed aberrantly expressed genes in endometriosis during the process of decidualization and normal window of implantation. Authors suggested that impaired decidualization and dysfunctional expression of genes related to Müllerian embryogenesis (like the downstream targets of *HOXA10*) could be critical to the development of endometriosis. Also, it was proposed that DNA methylation of specific genes could partly explain the link between early exposure to a detrimental fetal environment and an increased risk of developing endometriosis later in life [31]. Furthermore, it has been proposed that in utero exposure to endocrine disruptor bisphenol could be one potential cause triggering the abnormal fetal endometrial cell migration into ectopic location, as mice exposed in utero to bisphenol exhibited endometriosis-like phenotype [32]. One of the differentially methylated genes in our study was *AHRR*, which shows increased gene expression in fetal tissues exposed to environmental or even lower levels of bisphenol [33]. It has been proposed that developmental exposure to environmental toxins may induce irregular methylation patterns and thereby permanently alter the expression of AHRR [34]. The relevance of *AHRR* methylation to theory of endometrial origin of endometriosis is intriguing and worth further examination.

Some limitations of our study should be highlighted. Although analysed samples covered the whole menstrual cycle, the size of some study groups (e.g. M and P phases) was rather small. Moreover, the limited number of samples from particular menstrual cycle phases restricted the possibility to compare patients and controls from each phase separately. Furthermore, histological endometrial dating was available only for healthy volunteers from MS group and therefore, as the self-reported day of menstrual cycle is less accurate for phase dating, it could have some negative impacts on menstrual cycle phase-specific analysis.

## Conclusions

The results of this study demonstrated that endometrial DNA methylation profile of women with and without endometriosis was highly similar and thus, epigenetic modifications in endometria are probably not the primary source contributing to endometriosis development. Although some DMRs between patients with endometriosis and controls were found, the magnitude of the methylation differences was too small to enable discrimination between patients and controls. The findings of this study provide new knowledge about the normal epigenetic changes occurring across the menstrual cycle phases and accentuate the importance of considering normal cyclic epigenetic changes when looking for disease specific endometrial DNA methylation changes.

## Methods

### Ethics statement

The study was approved by the Research Ethics Committee of the University of Tartu (219/M-15) and written informed consent was obtained from all participants. Tissues from cases and controls from Oxford originated from the ENDOX study, which was approved by the NRES Committee South Central-Oxford Research Ethics Committee (09/H0604/58).

### Study subjects and tissue processing

Altogether, 31 patients and 24 disease-free women were recruited into the microarray study (Table 1). General characteristics, such as age and BMI were similar between patients and all controls (Student’s *t* test, *P* > 0.05).

Endometrial tissue samples were collected from 31 patients undergoing laparoscopy at the Tartu University Hospital Women’s Clinic, Elite Clinic (Tartu, Estonia, *n* = 24) and John Radcliffe Hospital (Oxford, UK, *n* = 7). In all cases, the diagnosis was histologically confirmed and disease severity was determined according to the American Society for Reproductive Medicine revised classification system [35]. All patients were in reproductive age, having received no hormonal medication during the previous 3 months before laparoscopic surgery and had a regular menstrual cycle (28 ± 5 days). Self-reported menstrual cycle day was used to estimate cycle phase.

Control group consisted of 24 disease-free women from whom 17 were self-reported healthy volunteers (Elite Clinic, Tartu and Nova Vita Clinic, Tallinn, Estonia) and seven were undergoing laparoscopy for pelvic pain, subfertility or tubal sterilisation and confirmed to be endometriosis free (Oxford control group). Healthy volunteers were all in reproductive age, had not used hormonal medication at least 3 months before the recruitment, had regular menstrual cycle (28 ± 5 days), had normal serum levels of progesterone, prolactin and testosterone, normal vaginal ultrasound, negative screening results for sexually transmitted diseases and no presence of endometriosis or polycystic ovary syndrome. Endometrial biopsies for the Estonian controls were collected under local anaesthesia, and menstrual cycle dating was confirmed by combining menstrual cycle history, luteinizing hormone (LH) peak (estimated by the BabyTime® hLH urine cassette, Pharmanova), vaginal ultrasound and by the histological evaluation of biopsy according to the Noyes’ criteria [36]. The menstrual cycle phases for Oxford controls were estimated according to their self-reported menstrual cycle day.

For validation study, an extended group of patients and controls from LS (*n* = 15) and MS phases (*n* = 14) was used, and in addition to endometrial samples from microarray study (*n* = 11), further endometrial samples from patients with endometriosis from LS phase (*n* = 4) and MS phase (*n* = 3) and healthy controls (*n* = 8) from LS phase and MS (*n* = 3) phase were collected (Table 1).

Endometrial biopsy samples from patients and controls were collected using an endometrial suction catheter (Pipelle, Laboratoire CCD).

### Pre-processing and normalisation of the methylation microarray data

DNA bisulfite treatment using EZ DNA Methylation kits (Zymo Research) and DNA hybridization to Infinium HumanMethylation 450K BeadChip were performed at USC Epigenome Center (Los Angeles, CA) according to the manufacturer’s specifications.

Microarray data from Estonian and Oxford datasets were combined for the data analysis. The raw intensity files were imported into the R statistical computing environment using Bioconductor package minfi [37]. The methylation value (beta value) for each probe was then computed into beta value using Illumina’s formula *M*/(*M* + *U* + 100), where *M* and *U* represent methylated and unmethylated signal intensities, respectively [38]. The delta beta (Δβ) value was calculated as difference in β-values between the two groups. Methylation values ranged from 0, fully unmethylated, to 1, fully methylated cytosine. Multiple quality control measures were then applied to filter out unwanted probes. Probes containing SNP sites (*n* = 65), probes with the detection *P* value >0.01 in more than one sample (*n* = 11055) and probes with the beadcount <3 in at least 5 % of the samples (*n* = 2074) were removed. The remaining 461,286 probes were normalised for adjusting type1 and type2 probes using Beta-Mixture Quantile (BMIQ) normalisation method [39]. Finally, the batch effect was corrected using ComBat normalisation method [40]. The preprocessing, quality control and batch effect analyses were performed using the Bioconductor ChAMP package [41]. Two Estonian samples and all Oxford samples were run as duplicates (technical replicates). The Pearson correlation coefficient was >0.99 for all replicates, confirming a good level of technical reproducibility. The duplicate beta values were averaged and used for further data analysis. PCA and unsupervised hierarchical clustering were performed as a part of quality control and to provide a visual overview of methylation differences between the samples. All analyses were performed using statistical computing environment R.

### Identification of DMRs

DMRs were identified using ‘seqlm’ package (https://github.com/raivokolde/seqlm) in the R environment, utilising MDL-based approach described earlier [18]. The Benjamini–Hochberg FDR was calculated for each probe, with an FDR corrected *P* value <0.05 used to define DMRs. The DMR analyses were performed to assess the differences between (i) endometria of healthy and endometriosis patients and (ii) menstrual cycle phases. In order to get optimal DMRs, we limited our search in regions where distance between at least three consecutive probes was ≤500 bp. Venn analysis, to determine overlaps between DMR genes, was performed using the web-based program VENNY 2.0 (http://bioinfogp.cnb.csic.es/tools/venny/).

### Validation of methylation array data by direct bisulfite sequencing

Four CpGs with differential methylation, two from *CST11* gene (cg06197930, cg12480562), one from *PI3* gene (cg19931348) and one from *SLC43A3* gene (cg13046608) were selected for validation analysis. Bisulfite modification of the endometrial DNA samples (500 ng each) was carried out with the EZ DNA Methylation-Gold™ kit (Zymo Research) according to the manufacturer’s specifications. PCR primers for the bisulfite-treated DNA were designed using MethPrimer [42]. PCR conditions and list of primers are provided in Additional file 8. The sequencing results were analysed as described in [43] and using Mutation Surveyor software (Softgenetics, State College, PA, USA).

### Functional enrichment analysis

A web-based tool g: Profiler was utilised to query genes from DMRs for GO category and KEGG (Kyoto Encyclopaedia of Genes and Genomes) pathway enrichment (http://biit.cs.ut.ee/gprofiler/) [44]. The FDR *P* value <0.05 was considered statistically significant.

## Availability of supporting data

The datasets supporting the results of this article have been deposited at NCBI Gene Expression Omnibus data repository with accession number GSE73950.

## Additional files

Additional file 1:
**Principal component analysis describing DNA methylation data across all studied endometrial samples.** The large dots and triangles mark overlapping samples. (TIF 852 kb) Additional file 2:
**Differentially methylated regions between women with endometriosis and healthy women.** The methylation data of all patients was compared to controls. Menstrual cycle day has not been taken into account. (XLSX 15 kb) Additional file 3:
**Differentially methylated regions between different menstrual cycle phases.** The methylation data of each menstrual cycle phase was compared to other phases. (XLSX 2676 kb) Additional file 4:
**Functional annotation clustering of hypo- and hypermethylated genes in endometrial tissue using g:profiler bioinformatics tool.** The complete lists of DMRs between different menstrual cycle phases was used to create the lists of Gene Ontology terms. (XLSX 27 kb) Additional file 5:
**Pathway analysis of hypo- and hypermethylated genes in endometrial tissue using g:profiler bioinformatics tool.** The complete lists of DMRs between different menstrual cycle phases was used to create the list of Kyoto Encyclopedia of Genes and Genomes (KEGG) pathways. (XLSX 12 kb) Additional file 6:
**Venn diagrams of differentially methylated genes.** Diagrams show the total number of hypo- and hypermethylated genes identified in each comparison. (TIF 3142 kb) Additional file 7:
**Menstrual cycle phase specific genes.** The list of late-secretory and menstrual phase specific hyper- and hypomethylated genes. (XLSX 28 kb) Additional file 8:
**PCR primers used in the methylation validation analysis.** PCR primers for the bisulfite-treated DNA were designed using MethPrimer[42]. (XLSX 9 kb)

## References

[1] Slieker RC, Bos SD, Goeman JJ, Bovee JV, Talens RP, van der Breggen R (2013). Identification and systematic annotation of tissue-specific differentially methylated regions using the Illumina 450k array. Epigenetics Chromatin.

[2] Muangsub T, Samsuwan J, Tongyoo P, Kitkumthorn N, Mutirangura A (2014). Analysis of methylation microarray for tissue specific detection. Gene.

[3] Xue Q, Xu Y, Yang H, Zhang L, Shang J, Zeng C (2014). Methylation of a novel CpG island of intron 1 is associated with steroidogenic factor 1 expression in endometriotic stromal cells. Reprod Sci.

[4] Wu Y, Strawn E, Basir Z, Halverson G, Guo SW (2006). Promoter hypermethylation of progesterone receptor isoform B (PR-B) in endometriosis. Epigenetics.

[5] Xue Q, Lin Z, Cheng YH, Huang CC, Marsh E, Yin P (2007). Promoter methylation regulates estrogen receptor 2 in human endometrium and endometriosis. Biol Reprod.

[6] Lu H, Yang X, Zhang Y, Lu R, Wang X (2013). Epigenetic disorder may cause downregulation of HOXA10 in the eutopic endometrium of fertile women with endometriosis. Reprod Sci.

[7] Fambrini M, Sorbi F, Bussani C, Cioni R, Sisti G, Andersson KL (2013). Hypermethylation of HOXA10 gene in mid-luteal endometrium from women with ovarian endometriomas. Acta Obstet Gynecol Scand.

[8] Andersson KL, Bussani C, Fambrini M, Polverini V, Taddei GL, Gemzell-Danielsson K et al. DNA methylation of HOXA10 in eutopic and ectopic endometrium. Hum Reprod. 2014. doi:10.1093/humrep/deu161. 10.1093/humrep/deu161 24963168

[9] Szczepanska M, Wirstlein P, Skrzypczak J, Jagodzinski PP (2012). Expression of HOXA11 in the mid-luteal endometrium from women with endometriosis-associated infertility. Reprod Biol Endocrinol..

[10] Wang D, Chen Q, Zhang C, Ren F, Li T (2012). DNA hypomethylation of the COX-2 gene promoter is associated with up-regulation of its mRNA expression in eutopic endometrium of endometriosis. Eur J Med Res..

[11] Izawa M, Taniguchi F, Uegaki T, Takai E, Iwabe T, Terakawa N (2011). Demethylation of a nonpromoter cytosine-phosphate-guanine island in the aromatase gene may cause the aberrant up-regulation in endometriotic tissues. Fertil Steril.

[12] Borghese B, Barbaux S, Mondon F, Santulli P, Pierre G, Vinci G (2010). Research resource: genome-wide profiling of methylated promoters in endometriosis reveals a subtelomeric location of hypermethylation. Mol Endocrinol.

[13] Yamagata Y, Nishino K, Takaki E, Sato S, Maekawa R, Nakai A (2014). Genome-wide DNA methylation profiling in cultured eutopic and ectopic endometrial stromal cells. PLoS One.

[14] Dyson MT, Roqueiro D, Monsivais D, Ercan CM, Pavone ME, Brooks DC (2014). Genome-wide DNA methylation analysis predicts an epigenetic switch for GATA factor expression in endometriosis. PLoS Genet.

[15] Naqvi H, Ilagan Y, Krikun G, Taylor HS. Altered genome-wide methylation in endometriosis. Reprod Sci. 2014. doi:10.1177/1933719114532841. 10.1177/1933719114532841 PMC5933183 24784717

[16] Bouquet de Joliniere J, Ayoubi JM, Lesec G, Validire P, Goguin A, Gianaroli L (2012). Identification of displaced endometrial glands and embryonic duct remnants in female fetal reproductive tract: possible pathogenetic role in endometriotic and pelvic neoplastic processes. Front Physiol.

[17] Signorile PG, Baldi A (2010). Endometriosis: new concepts in the pathogenesis. Int J Biochem Cell Biol.

[18] Lokk K, Modhukur V, Rajashekar B, Martens K, Magi R, Kolde R (2014). DNA methylome profiling of human tissues identifies global and tissue-specific methylation patterns. Genome Biol.

[19] Zhang B, Zhou Y, Lin N, Lowdon RF, Hong C, Nagarajan RP (2013). Functional DNA methylation differences between tissues, cell types, and across individuals discovered using the M&M algorithm. Genome Res.

[20] Houshdaran S, Zelenko Z, Irwin JC, Giudice LC (2014). Human endometrial DNA methylome is cycle-dependent and is associated with gene expression regulation. Mol Endocrinol.

[21] Rogers PA, D’Hooghe TM, Fazleabas A, Giudice LC, Montgomery GW, Petraglia F (2013). Defining future directions for endometriosis research: workshop report from the 2011 World Congress of Endometriosis in Montpellier. France. Reprod Sci..

[22] Edgar R, Tan PP, Portales-Casamar E, Pavlidis P (2014). Meta-analysis of human methylomes reveals stably methylated sequences surrounding CpG islands associated with high gene expression. Epigenetics Chromatin.

[23] Wee EJ, Ha Ngo T, Trau M (2015). A simple bridging flocculation assay for rapid, sensitive and stringent detection of gene specific DNA methylation. Sci Rep..

[24] Tamaresis JS, Irwin JC, Goldfien GA, Rabban JT, Burney RO, Nezhat C et al. Molecular classification of endometriosis and disease stage using high-dimensional genomic data. Endocrinology. 2014:en20141490. doi:10.1210/en.2014-1490. 10.1210/en.2014-1490 PMC4239429 25243856

[25] Furukawa J, Inoue K, Maeda J, Yasujima T, Ohta K, Kanai Y (2015). Functional identification of SLC43A3 as an equilibrative nucleobase transporter involved in purine salvage in mammals. Sci Rep..

[26] Stuart RO, Pavlova A, Beier D, Li Z, Krijanovski Y, Nigam SK (2001). EEG1, a putative transporter expressed during epithelial organogenesis: comparison with embryonic transporter expression during nephrogenesis. Am J Physiol Renal Physiol.

[27] Talbi S, Hamilton AE, Vo KC, Tulac S, Overgaard MT, Dosiou C (2006). Molecular phenotyping of human endometrium distinguishes menstrual cycle phases and underlying biological processes in normo-ovulatory women. Endocrinology.

[28] Ponnampalam AP, Weston GC, Trajstman AC, Susil B, Rogers PA (2004). Molecular classification of human endometrial cycle stages by transcriptional profiling. Mol Hum Reprod.

[29] Celik O, Unlu C, Otlu B, Celik N, Caliskan E (2015). Laparoscopic endometrioma resection increases peri-implantation endometrial HOXA-10 and HOXA-11 mRNA expression. Fertil Steril.

[30] Wu Y, Halverson G, Basir Z, Strawn E, Yan P, Guo SW (2005). Aberrant methylation at HOXA10 may be responsible for its aberrant expression in the endometrium of patients with endometriosis. Am J Obstet Gynecol.

[31] Kobayashi H, Iwai K, Niiro E, Morioka S, Yamada Y (2014). Fetal programming theory: implication for the understanding of endometriosis. Hum Immunol.

[32] Signorile PG, Spugnini EP, Mita L, Mellone P, D’Avino A, Bianco M (2010). Pre-natal exposure of mice to bisphenol A elicits an endometriosis-like phenotype in female offspring. Gen Comp Endocrinol.

[33] Nishizawa H, Imanishi S, Manabe N (2005). Effects of exposure in utero to bisphenol a on the expression of aryl hydrocarbon receptor, related factors, and xenobiotic metabolizing enzymes in murine embryos. J Reprod Dev.

[34] Aragon AC, Kopf PG, Campen MJ, Huwe JK, Walker MK (2008). In utero and lactational 2,3,7,8-tetrachlorodibenzo-p-dioxin exposure: effects on fetal and adult cardiac gene expression and adult cardiac and renal morphology. Toxicol Sci.

[35] Revised American Society for Reproductive Medicine classification of endometriosis: 1996 (1997). Fertility and sterility.

[36] Noyes RW, Hertig AT, Rock J (1975). Dating the endometrial biopsy. Am J Obstet Gynecol.

[37] Aryee MJ, Jaffe AE, Corrada-Bravo H, Ladd-Acosta C, Feinberg AP, Hansen KD (2014). Minfi: a flexible and comprehensive Bioconductor package for the analysis of Infinium DNA methylation microarrays. Bioinformatics.

[38] Bibikova M, Barnes B, Tsan C, Ho V, Klotzle B, Le JM (2011). High density DNA methylation array with single CpG site resolution. Genomics.

[39] Teschendorff AE, Marabita F, Lechner M, Bartlett T, Tegner J, Gomez-Cabrero D (2013). A beta-mixture quantile normalization method for correcting probe design bias in Illumina Infinium 450k DNA methylation data. Bioinformatics.

[40] Johnson WE, Li C, Rabinovic A (2007). Adjusting batch effects in microarray expression data using empirical Bayes methods. Biostatistics.

[41] Morris AP, Voight BF, Teslovich TM, Ferreira T, Segre AV, Steinthorsdottir V (2012). Large-scale association analysis provides insights into the genetic architecture and pathophysiology of type 2 diabetes. Nat Genet.

[42] Li LC, Dahiya R (2002). MethPrimer: designing primers for methylation PCRs. Bioinformatics.

[43] Parrish RR, Day JJ, Lubin FD (2012). Direct bisulfite sequencing for examination of DNA methylation with gene and nucleotide resolution from brain tissues. Curr Protoc Neurosci.

[44] Reimand J, Kull M, Peterson H, Hansen J, Vilo J (2007). g:Profiler—a web-based toolset for functional profiling of gene lists from large-scale experiments. Nucleic Acids Res.

