# Supplementary figures and images for: The influence of menstrual cycle and endometriosis on endometrial methylome
Source: Clin Epigenetics. 2016 Jan 12;8:2. doi: 10.1186/s13148-015-0168-z (PMC4710036; doi:10.1186/s13148-015-0168-z)

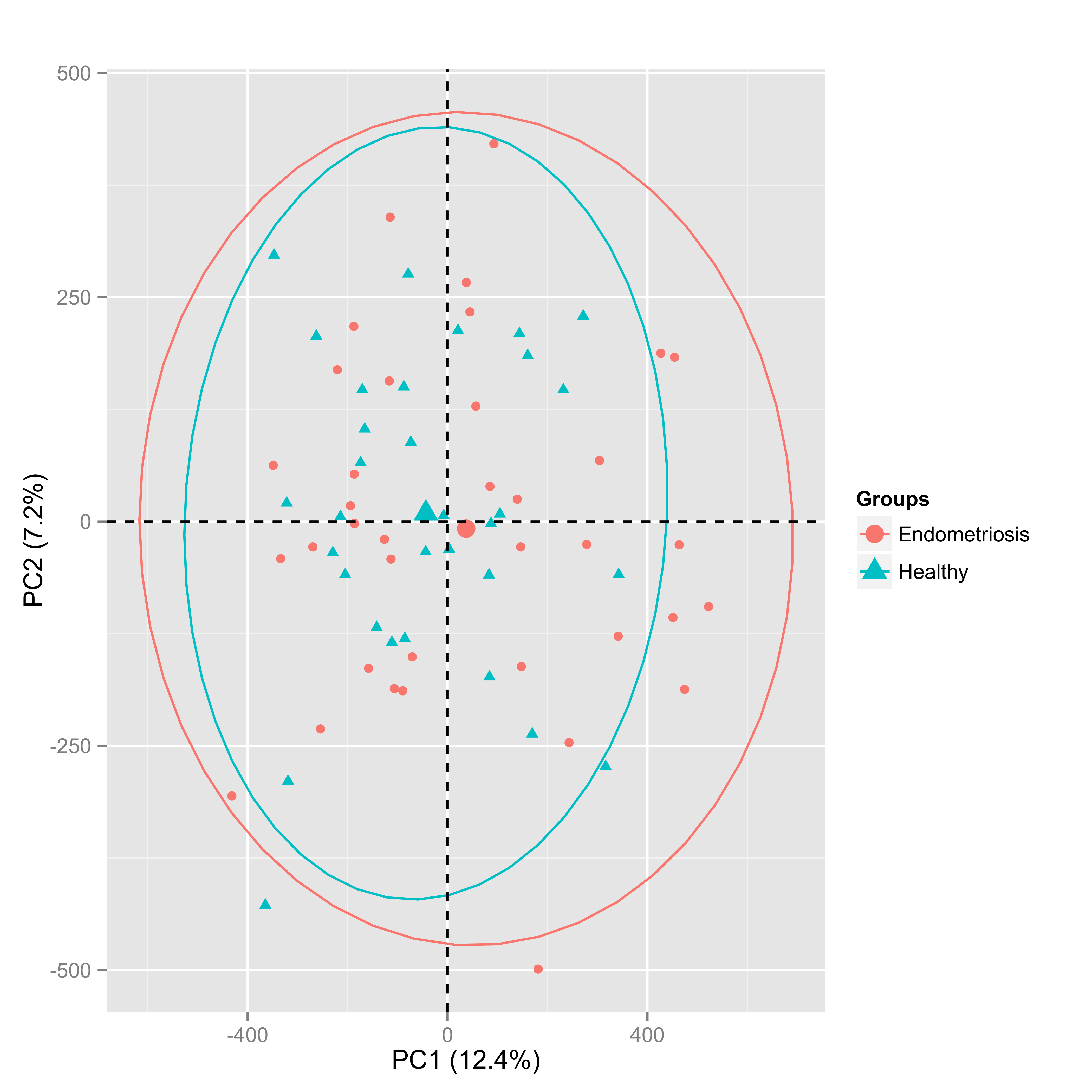

Supplement: Additional file 1: — Principal component analysis describing DNA methylation data across all studied endometrial samples. The large dots and triangles mark overlapping samples. (TIF 852 kb) [file 13148_2015_168_MOESM1_ESM.tif]

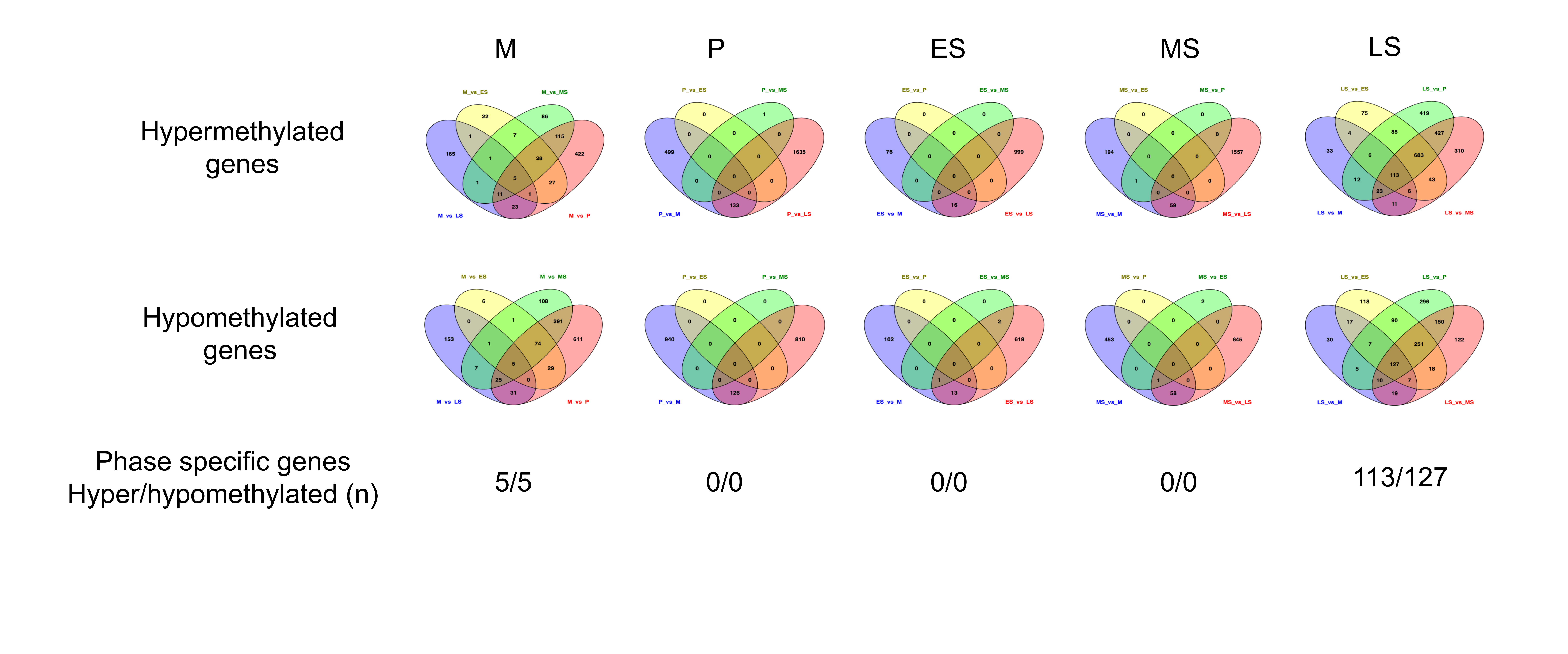

Supplement: Additional file 6: — Venn diagrams of differentially methylated genes. Diagrams show the total number of hypo- and hypermethylated genes identified in each comparison. (TIF 3142 kb) [file 13148_2015_168_MOESM6_ESM.tif]
